# FN1 from cancer-associated fibroblasts orchestrates pancreatic cancer metastasis via integrin-PI3K/AKT signaling

**DOI:** 10.3389/fonc.2025.1595523

**Published:** 2025-07-03

**Authors:** Xianguang Zhu, Yanwei Li, Huifang Liu, Zheng Xiao

**Affiliations:** ^1^ Department of Clinical Laboratory, Henan Provincial People’s Hospital, People’s Hospital of Zhengzhou University, and People’s Hospital of Henan University, Zhengzhou, Henan, China; ^2^ Department of Pathology, The Fifth Affiliated Hospital of Zhengzhou University, Zhengzhou, Henan, China

**Keywords:** fibronectin 1, pancreatic cancer, metastasis, integrin-PI3K/AKT signaling axis, cancer-associated fibroblasts

## Abstract

**Objective:**

The metastasis of pancreatic ductal adenocarcinoma (PDAC) is closely linked to the remodeling of cancer-associated fibroblasts (CAFs) within the tumor microenvironment (TME), though the precise molecular mechanisms remain unclear. This study aims to elucidate the role of CAFs in PDAC metastasis.

**Methods:**

We integrated transcriptomic (GSE183795) and single-cell sequencing data (GSE156405) to screen for core genes associated with PDAC. *In vitro* co-culture models, functional assays (Transwell migration, Western blotting, qRT-PCR), and clinical data analysis were employed.

**Results:**

Multi-omics analysis identified FN1 as a pivotal hub gene in the PI3K pathway, highly expressed in metastatic CAF subsets. *In vitro* experiments confirmed that FN1 activates the PI3K/AKT pathway through integrin receptors, influencing cell invasion and the immune microenvironment. Combined inhibition of the PI3K/AKT pathway and integrins synergistically suppressed tumor invasion. Clinical data showed that high FN1 expression correlated with shortened patient survival and an immunosuppressive microenvironment (M2 macrophage/Treg cell infiltration).

**Conclusion:**

FN1 directly drives PDAC metastasis via the integrin-PI3K/AKT axis and indirectly promotes a “cold tumor” microenvironment by recruiting immunosuppressive cells. This dual role of FN1 enhances our understanding of CAFs heterogeneity and offers novel therapeutic strategies for PDAC.

## 1 Introduction

Pancreatic cancer is one of the leading causes of cancer-related deaths globally, with a five-year survival rate of less than 10%. Over 80% of patients have local or distant metastasis at diagnosis, and more than 90% of pancreatic malignancies are classified as pancreatic ductal adenocarcinoma (PDAC) (1). Despite recent breakthroughs in targeted therapies (such as KRAS G12C inhibitors) and immunotherapies (such as PD-1/PD-L1 inhibitors) in some solid tumors, pancreatic cancer remains a “therapeutic desert” due to its highly fibrotic tumor microenvironment (TME) and complex cellular interaction networks (2). The core of this therapeutic dilemma lies in the dynamic remodeling of the TME: Cancer-associated fibroblasts (CAFs) secrete extracellular matrix (ECM) components and soluble factors, not only forming physical barriers to impede drug penetration but also directly promoting tumor cell invasion, metastasis, and immune escape through paracrine signaling (3).

The TME of PDAC is a complex ecosystem composed of CAFs, immune cells, endothelial cells, and other components (4). Among them, CAFs are one of the most prominent and plastic cell types and are considered key players with multiple tumor-promoting functions. CAFs in cancer are capable of producing ECM components that act as physical barriers, increasing tissue stiffness, and subsequently impairing processes such as drug delivery (5).

Advances in single-cell transcriptome research have revealed significant heterogeneity among CAFs (6). For instance, myofibroblastic CAFs (myCAFs) highly express α-SMA (*ACTA2*) and collagens (such as *COL1A1*), driving increased tumor stiffness through ECM crosslinking and mechanical signaling. In contrast, inflammatory CAFs (iCAFs) secrete cytokines such as IL-6 and CXCL12, recruiting immunosuppressive cells and maintaining stem cell properties (7). Although myCAFs are widely considered closely associated with metastasis, the specific ECM components they secrete and how they synergize with intracellular signaling pathways in tumor cells remain unclear. Notably, the phosphatidylinositol 3-kinase-protein kinase B (PI3K/AKT) pathway is frequently activated in pancreatic cancer (8), but its upstream regulatory mechanisms—particularly how ECM components trigger this pathway through membrane receptors such as integrins—have not been systematically analyzed.

Fibronectin 1 (FN1), a core component of the ECM, has been reported in various cancers to activate the PI3K/AKT and FAK pathways through integrin receptors, promoting cell migration, invasion, and chemotherapy resistance (9, 10). In pancreatic cancer, studies have found that FN1, encoding fibronectin, serves as a key signal transduction gene for therapeutic intervention in pancreatic cancer and can serve as a potential therapeutic and diagnostic biomarker for metastatic pancreatic tumors (11). Recent single-cell studies suggest a potential role for myCAFs in liver metastasis and recurrence in metastatic colorectal cancer, specifically through paracrine signaling that remodels the ECM, generates FN1, BGN, and other ECM components to promote liver metastasis of colorectal cancer (12). However, whether FN1 directly drives pancreatic cancer metastasis through the integrin-PI3K/AKT axis and whether it simultaneously participates in the remodeling of the immune microenvironment remain unanswered questions. In addition, the synergistic effects of FN1 with other ECM components (such as *COL1A1*, *THBS2*) and their clinical significance await further exploration.

Complicating matters further, the immune microenvironment of pancreatic cancer is characterized by high immunosuppression, with infiltration of M2 macrophages and regulatory T cells (Tregs) significantly correlating with poor patient survival (13). Studies have shown that ECM components (such as hyaluronic acid) can inhibit T-cell infiltration through physical barriers or signaling conduction (14), but whether FN1 mediates immune escape through similar mechanisms is unclear. This dual role of ECM-immune interactions may provide new targets for pancreatic cancer treatment, but the relevant mechanistic research is still in its infancy.

Based on the above background, this study proposes the following scientific hypotheses: metastatic CAFs promote invasion by secreting FN1 to activate the integrin-PI3K/AKT signaling axis in tumor cells; simultaneously, FN1 shapes an immunosuppressive microenvironment by recruiting M2 macrophages and Tregs, indirectly accelerating the metastatic process. Through the integration of multi-omics data, *in vitro* coculture models, clinical cohort analysis, and mechanistic exploration, this study aims to: (1) analyze the core role of FN1 in CAF-tumor cell interactions; (2) elucidate the molecular mechanisms by which FN1 regulates the PI3K/AKT pathway and the immune microenvironment; (3) evaluate the translational potential of FN1 as a prognostic biomarker and therapeutic target.

## 2 Materials and methods

### 2.1 Bioinformatics analysis

The transcriptome dataset GSE183795 containing pancreatic cancer and adjacent non-cancerous tissues was downloaded from the Gene Expression Omnibus (GEO, https://www.ncbi.nlm.nih.gov/geo/). Differential gene expression analysis was conducted using the limma package (v3.62.2) in R language (v4.4.2), with a screening threshold of |log2FC| ≥ 1 and *p <* 0.05. Significantly different genes were visualized through a volcano plot (generated using the ggplot2 package, v3.5.1), and the expression patterns of the top 50 differentially expressed genes were displayed in a heatmap (generated using the pheatmap package, v1.0.12). Functional enrichment analysis was performed using the clusterProfiler package (v4.14.4) based on KEGG pathways and GO terms (FDR < 0.05). Core genes in the P13K/AKT signaling pathway were screened using the CytoHubba plugin (MCC algorithm) in Cytoscape software (v3.9.1), selecting the top 10 genes with the highest interaction scores.

The single-cell RNA sequencing dataset GSE156405 (samples from primary pancreatic cancer tumors (stiu) and liver metastases (meta)) was processed using the Seurat package (v5.2.0). Quality control filtering criteria included ≥200 genes detected per cell and a mitochondrial gene proportion <20%. The data were normalized (LogNormalize), subjected to principal component analysis (PCA), and dimensionally reduced using Uniform Manifold Approximation and Projection (UMAP) before clustering (resolution = 0.5). Cell type annotation was based on classic marker genes. Differential gene analysis was conducted using the FindMarkers function (Wilcoxon test, log2FC > 0.25), and enrichment analysis focused on KEGG pathways. Fibroblast subpopulations were re-clustered after subset extraction (resolution = 0.3), and CAFs were defined by markers such as ACTA2 and FAP.

### 2.2 *In vitro* coculture model construction

Cell Source and Induction: Immortalized human pancreatic CAFs were purchased from Saios (Cat# CL-417h) and routinely cultured in DMEM medium (Corning, USA, Cat# 10-013-CV) containing 10% fetal bovine serum (FBS, Gibco, USA, Cat# 16000044). To activate CAFs, TGF-β1 (PeproTech, USA, Cat# 100-21C, 10 ng/mL) was added for 48 hours of treatment.

Co-culture system: (1) The activated CAFs were co-cultured with pancreatic cancer cells PANC-1 (ATCC, Cat# CRL-1469) or human primary pancreatic cancer cells BXPC-3 (Procell, China, Cat# CL-0042) at a ratio of 1:2 in Transwell inserts (Corning, USA, Cat# 3422). Specifically, PANC-1 or BXPC-3 cells were seeded in the upper chambers, while the lower chambers were filled with CAF-conditioned medium (containing supernatant induced by TGF-β1).

### 2.3 Migration and invasion experiments

In the migration experiment, PANC-1 cells (5×10^4^/well) were seeded in the upper chamber of a Transwell insert, and CAFs conditioned medium was added to the lower chamber. After 48 hours of incubation, the cells were fixed with 4% paraformaldehyde (Sigma-Aldrich, USA, Cat# P6148) and stained with 0.1% crystal violet (Sigma-Aldrich, USA, Cat# C0775). The number of migrated cells was counted using ImageJ software (v1.53). For the invasion experiment, the Transwell insert was pre-coated with Matrigel (BD Biosciences, USA, Cat# 354230), following the same procedure as the migration assay, PANC-1 cells or BXPC-3 cells (5×10^4^ cells/well) were seeded into the upper chambers of the Transwell inserts. CAF-conditioned medium was added to the lower chambers. After 48 hours of incubation, the cells were fixed with 4% paraformaldehyde (Sigma-Aldrich, USA, Cat# P6148) and stained with 0.1% crystal violet (Sigma-Aldrich, Cat# C0775).

### 2.4 Real-time quantitative PCR

Total RNA was extracted from cells or tissues using TRIzol reagent (Invitrogen) according to the manufacturer’s instructions for detecting mRNA. Equal amounts of RNA were reverse transcribed into cDNA using the SuperScript Reverse Transcriptase Kit (Thermo Fisher Scientific). Total cDNA was amplified and analyzed in the Fast Real-time PCR-7500 system using SYBR Green PCR Master Mix (Thermo Fisher Scientific). The thermal cycling conditions were as follows: 95°C for 10 min, followed by 40 cycles of 95°C for 10 s, 55°C for 10 s, and 72°C for 30 s. Amplification was performed using Exicycler 96 (Bioneer™ Corporation), and relative expression levels were calculated based on the 2^−ΔΔCt^ method. All primer sequences are shown in **Table 1**.

**Table 1:** Sequences of primers.

Name	Sequences (5′-3′)
RT-ITGA2-F	CCTACAATGTTGGTCTCCCAGA
RT-ITGA2-R	AGTAACCAGTTGCCTTTTGGATT
RT-ITGB4-F	GCAGCTTCCAAATCACAGAGG
RT-ITGB4-R	CCAGATCATCGGACATGGAGTT
RT-ITGA3-F	TCAACCTGGATACCCGATTCC
RT-ITGA3-R	GCTCTGTCTGCCGATGGAG
RT-GAPDH-F	TCATGGGTGTGAACCATGAGAA
RT-GAPDH-R	GGCATGGACTGTGGTCATGAG

### 2.5 Western Blot analysis

The experiment was divided into a control group (ITG-Con) and an integrin inhibitor group (ITG-Inh). DMSO solvent (Sigma-Aldrich, Cat# D2650) or integrin inhibitor (ITG-Inh, Cilengitide 10 μM) was added to the coculture system, respectively. After 48 hours of coculture, PANC-1 cells and BXPC-3 cells were collected, and total protein was extracted using RIPA lysis buffer (Beyotime, China, Cat# P0013B). Protein concentration was determined using the BCA method (Thermo Fisher, USA, Cat# 23225). Protein samples (20 μg) were separated by 10% SDS-PAGE and transferred to PVDF membranes (Millipore, USA, Cat# IPVH00010). After blocking with 5% skimmed milk, the membranes were incubated with primary antibodies against p-AKT (Cell Signaling, USA, Cat#4060, rabbit, 1:1000), AKT (Cell Signaling, USA, Cat#9272, rabbit, 1:1000), p-PI3K (Cell Signaling, USA, Cat#4228, rabbit, 1:1000), PI3K (Cell Signaling, USA, Cat#4292, rabbit, 1:1000), and β-actin (Proteintech, USA, Cat# 60008-1-Ig, mouse, 1:5000). Subsequently, the membranes were incubated with Goat Anti-Rabbit secondary antibody (HRP-labeled, Proteintech, Cat# SA00001-2, 1:5000) and Goat Anti-Mouse secondary antibody (HRP-labeled, Proteintech, Cat# SA00001-1, 1:5000). Chemiluminescent detection was performed using ECL reagent (Thermo Fisher, USA, Cat# 32106), and the grayscale values were quantified using ImageJ software. Each group was repeated three times.

### 2.6 ELISA detection of secreted factors

FN1 neutralizing antibody (FN1-Ab) was purchased from R&D Systems (Cat# MAB19182). The antibody was used at a concentration of 10 μg/mL to neutralize FN1 in the co-culture system. For the control group (FN1-Con), an equivalent volume of phosphate-buffered saline (PBS) was added to the co-culture system instead of the FN1-Ab. Coculture supernatants were collected to detect the levels of IL-6 (R&D Systems, Cat# D6050), IL-8 (R&D Systems, Cat# D8000C), MMP2 (R&D Systems, Cat# DMP200), and FN1 (R&D Systems, Cat# DF1910) according to the manufacturer’s instructions provided with the ELISA kits.

### 2.7 Statistical analysis

All statistical analyses were performed using GraphPad Prism 10 software. For comparisons among three or more groups, one-way analysis of variance (One-way ANOVA) combined with Tukey’s multiple comparisons test was employed. For comparisons between two groups, Student’s t-test was used. The significance levels were set as *p < 0.05, **p < 0.01, and ***p < 0.001, with all p-values representing the results of two-tailed tests. Each experiment was repeated at least three times to ensure the reproducibility and stability of the results.

## 3 Results

### 3.1 Transcriptome analysis revealed the central role of the ECM-PI3K pathway in pancreatic cancer metastasis

**Figure 1:**
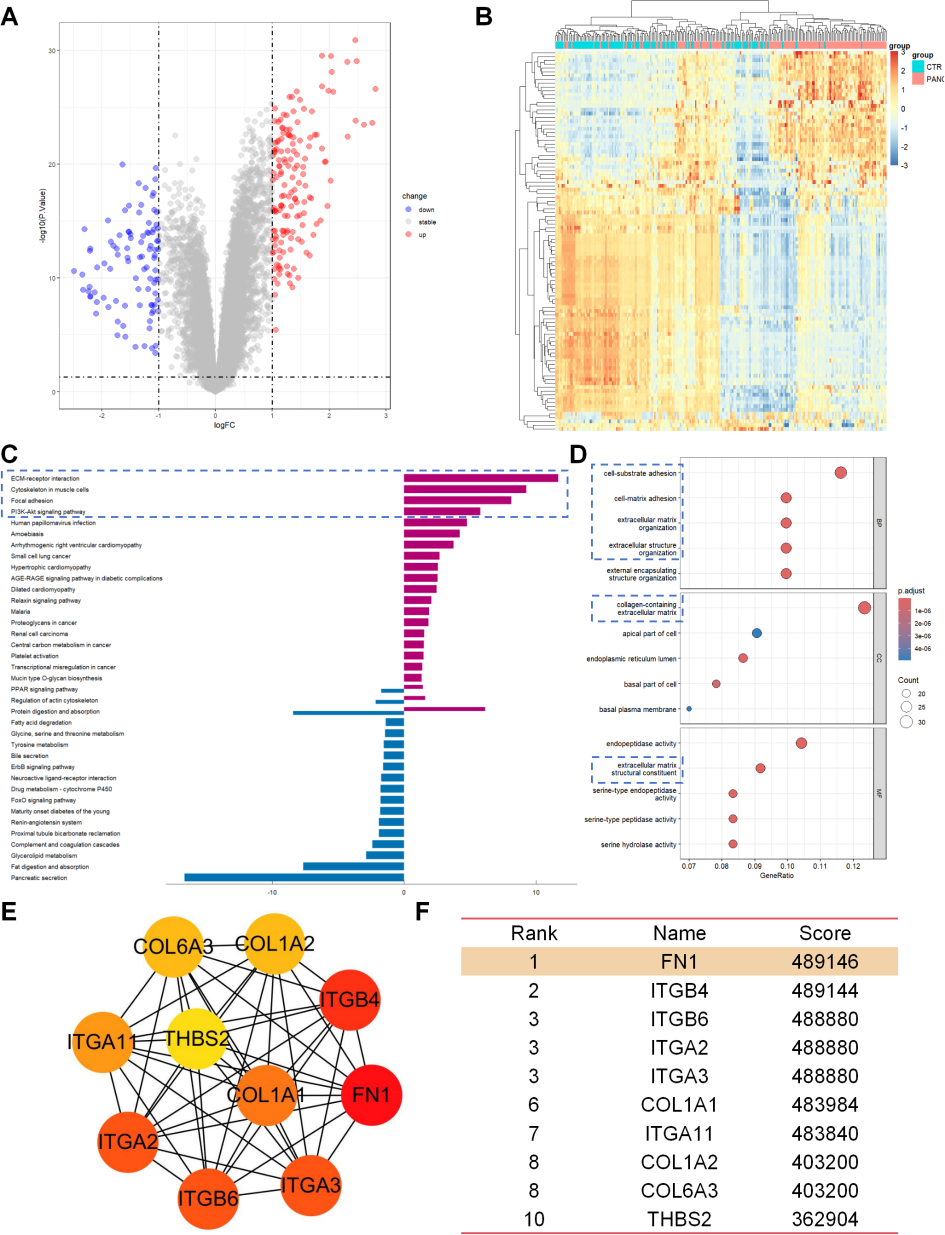
Transcriptome analysis revealed the central role of the ECM-PI3K pathway in pancreatic cancer metastasis. **(A)** Volcano plot of differentially expressed genes (DEGs), showing 248 DEGs between pancreatic cancer tissues (PANC) and adjacent normal tissues (CTR), with 153 genes upregulated and 95 genes downregulated. **(B)** Hierarchical clustering heatmap of the top 100 DEGs, demonstrating differences in gene expression patterns between PANC and CTR tissues. **(C)** Results of KEGG functional enrichment analysis, indicating that the DEGs are primarily involved in signaling pathways such as ECM-receptor interaction, Cytoskeleton in muscle cells, Focal adhesion, and P13K/AKT signaling pathway. **(D)** GO analysis results, showing significant enrichment of DEGs in processes related to adhesion and ECM. **(E, F)** CytoHubba analysis results, revealing FN1 as a core hub gene in the P13K/AKT signaling pathway.

Transcriptome analysis of the GSE183795 dataset revealed 248 differentially expressed genes (DEGs), including 153 upregulated genes and 95 downregulated genes (**Figure 1A**). Hierarchical clustering analysis of the top 100 DEGs indicated distinct gene expression patterns between pancreatic cancer tissues (PANC) and control (CTR) (**Figure 1B**). Kyoto Encyclopedia of Genes and Genomes (KEGG) functional enrichment analysis showed that these DEGs were primarily involved in ECM-receptor interaction, cytoskeleton in muscle cells, focal adhesion, and the PI3K/AKT signaling pathway (**Figure 1C**). Gene Ontology (GO) analysis further demonstrated that these genes were significantly enriched in processes related to adhesion and ECM (**Figure 1D**). Given the crucial roles of ECM-related changes and cell adhesion in pancreatic cancer metastasis, and the pivotal role of the PI3K/AKT pathway in various physiological processes such as cell growth, proliferation, and migration, the KEGG and GO results indicated significant enrichment of DEGs in ECM and cell adhesion-related pathways with potential links to the PI3K/AKT signaling pathway. To further explore the molecular mechanisms of pancreatic cancer metastasis and clarify the specific role and key genes of the PI3K/AKT pathway, we focused our research on the PI3K/AKT pathway. Among the genes related to the PI3K/AKT signaling pathway, FN1 emerged as the top-ranked hub gene based on CytoHubba analysis (**Figures 1E, F**). This finding suggests that FN1 may drive pancreatic cancer metastasis through the PI3K pathway.

### 3.2 Single-cell sequencing identifies specific expression patterns of PI3K-related genes including FN1 in fibroblasts of metastatic samples

**Figure 2:**
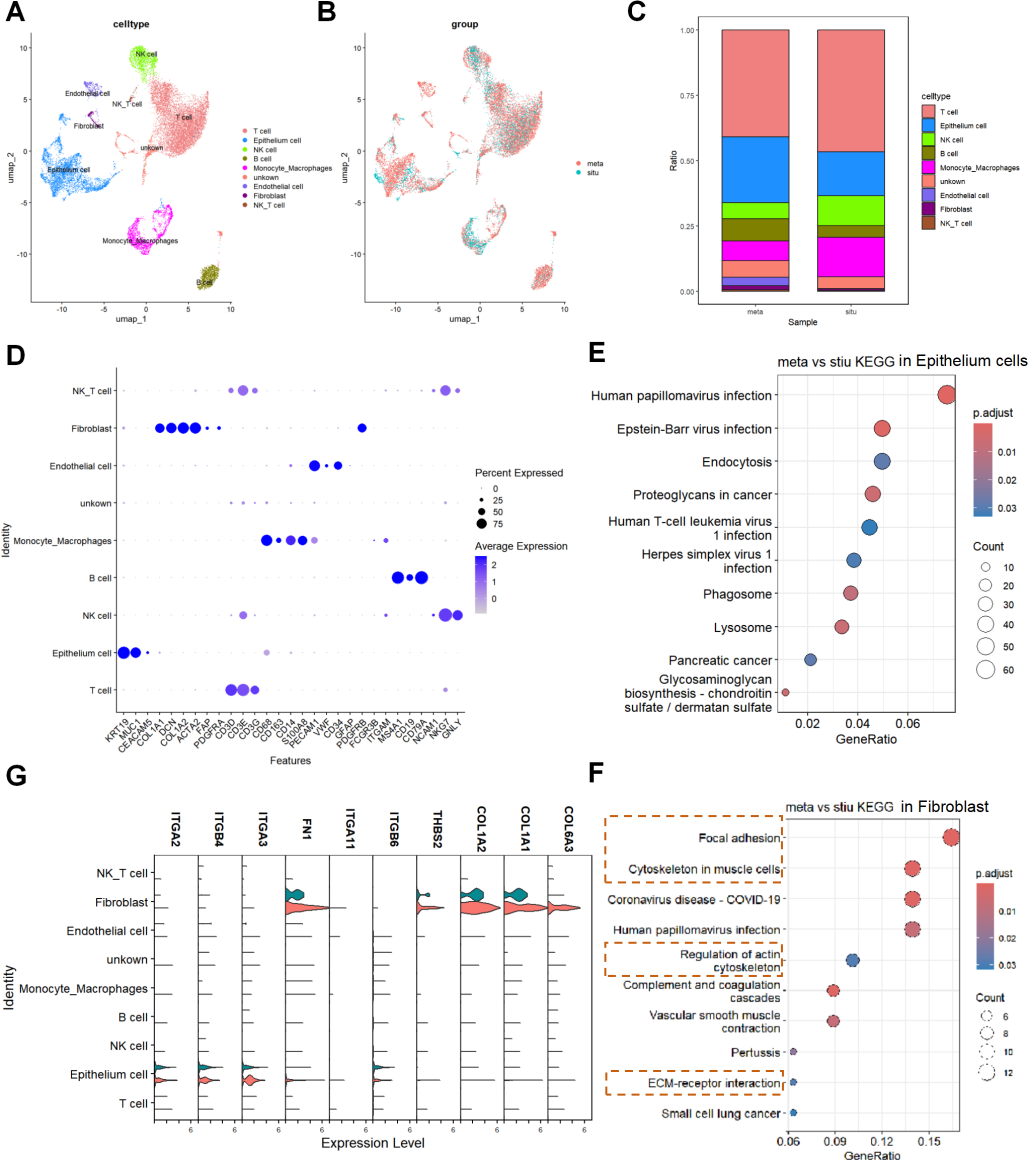
Single-cell sequencing identifies specific expression patterns of PI3K-related genes including FN1 in fibroblasts of metastatic samples. **(A)** UMAP plot displaying the sub-cell types in the primary pancreatic cancer (stiu) and liver metastasis (meta) samples from the single-cell RNA sequencing dataset GSE156405. **(B)** UMAP plot showing the expression patterns of different groups in primary pancreatic cancer (stiu) and liver metastasis (meta) samples. **(C)** Cell proportion plot illustrating the distribution of different cell types in stiu and meta samples. **(D)** Bubble plot representing the relationship between different sub-cell types and characteristic gene expression. The color of the bubbles ranges from white to blue, representing gene expression percentages of 0%, 25%, 50%, and 75%, respectively. The size of the bubbles indicates the average expression level, ranging from a minimum to a maximum representing average expression values from 0 to 2. **(E)** KEGG enrichment analysis of DEGs in alveolar epithelial cells. **(F)** KEGG enrichment analysis of DEGs in fibroblasts. **(G)** Violin plot showing the expression patterns of PI3K-related genes (FN1, THBS2, COL1A1, COL1A2, and COL6A3) in different cell types and groups in fibroblasts.

To validate the transcriptome results, we analyzed the single-cell RNA sequencing dataset GSE156405, which included samples from primary pancreatic cancer tumors (stiu) and liver metastases (meta), and identified the main cell types and proportions in both groups (**Figures 2A–D**). Comparative analysis revealed a significant increase in the proportion of fibroblasts in meta samples compared to stiu samples (**Figure 2C**). Pathway enrichment analysis of differentially expressed genes in fibroblasts from meta samples showed significant overlap with the transcriptome analysis results, particularly in terms of adhesion, cytoskeleton, and ECM (**Figure 2F**). In contrast, epithelial cells did not show similar enriched pathways as the transcriptome analysis (**Figure 2E**). Notably, PI3K-related genes including *FN1*, *THBS2*, *COL1A1*, *COL1A2*, and *COL6A3* were significantly upregulated in fibroblasts from metastatic samples (**Figure 2G**).

### 3.3 Core mechanisms of CAFs in pancreatic cancer metastasis

**Figure 3:**
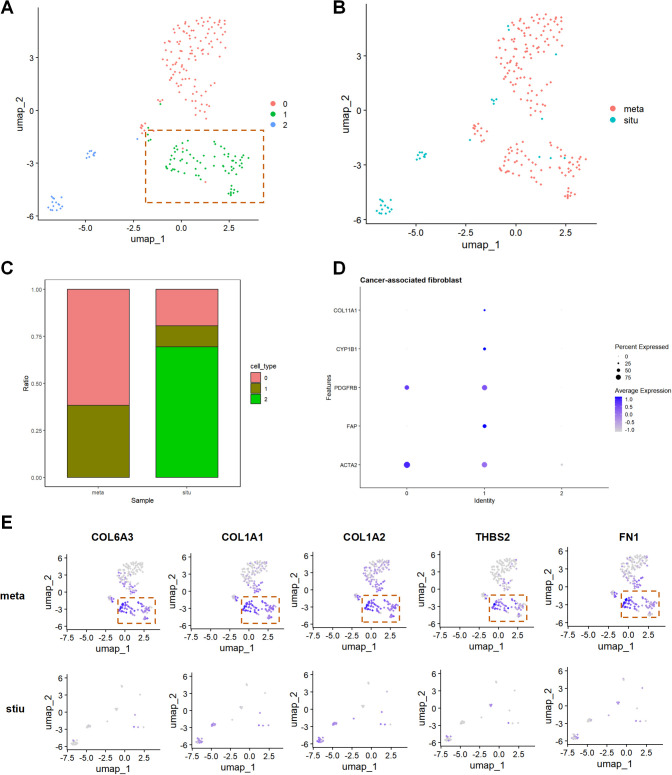
Core mechanisms of CAFs in pancreatic cancer metastasis. **(A)** UMAP plot displaying fibroblast subpopulations by cluster. **(B)** UMAP plot showing the distribution of fibroblast subpopulations by group. **(C)** Proportion plot illustrating the cell proportions by cell subpopulation and group. **(D)** Identification of the CAFs subpopulation among fibroblast subpopulations. **(E)** UMAP visualization analysis, displaying the expression patterns of FN1, THBS2, COL1A1, COL1A2, and COL6A3 in the stiu and meta groups.

Furthermore, re-clustering analysis of fibroblasts identified three distinct subpopulations, with subpopulation 1 being identified as CAFs based on high expression of genes such as ACTA2 and FAP (**Figures 3A–D**). Compared to stiu samples, the proportion of CAFs was significantly higher in meta samples (**Figure 3C**). UMAP visualization showed that FN1, THBS2, COL1A1, COL1A2, and COL6A3 were mainly expressed in CAFs from metastatic samples (**Figure 3E**). These results suggest that CAFs may play a crucial role in pancreatic cancer metastasis by upregulating ECM and PI3K/AKT related genes.

### 3.4 CAF-derived FN1 promotes invasion and migration of pancreatic cancer cells

**Figure 4:**
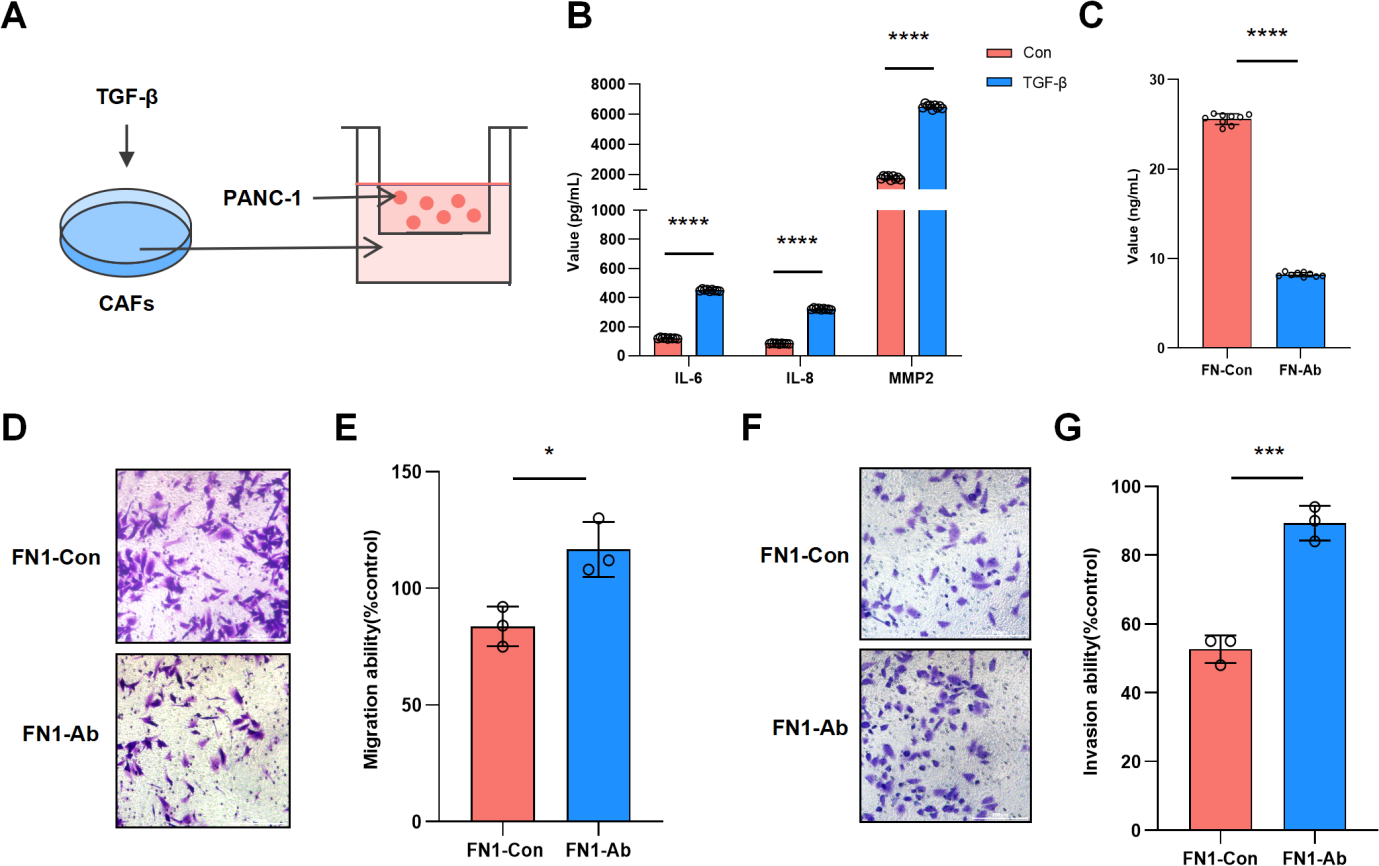
CAF-derived FN1 promotes invasion and migration of pancreatic cancer cells. **(A)** Schematic diagram of the CAFs-PANC1 co-culture model. **(B)** ELISA results showing changes in IL-6, IL-8, and MMP2 levels secreted by CAFs before and after TGF-β induction. **(C)** ELISA results demonstrating changes in FN1 levels secreted by CAFs before and after treatment with FN1 neutralizing antibody (FN1-Ab). **(D)** Transwell migration assay showing changes in the number of migrated cells in the FN1-Ab group compared to the control group. **(E)** Statistics of migration rates from the Transwell migration assay. **(F)** Transwell invasion assay showing changes in the number of invading cells in the FN1-Ab group compared to the control group. **(G)** Statistics of invasion rates from the Transwell invasion assay.

In the CAFs-PANC1 co-culture model (**Figure 4A**), ELISA results showed significant increases in the levels of IL-6, IL-8, and MMP2 (*p <* 0.0001) secreted by CAFs after TGF-β induction, confirming the activation state of CAFs (**Figure 4B**). After adding an FN1 neutralizing antibody (FN1-Ab) to the TGF-β-induced co-culture model, ELISA results showed a significant decrease in FN1 secretion by CAFs compared to the control group (FN1-Con) (*p <* 0.0001) (**Figure 4C**). Transwell migration and invasion assays showed a reduction in the number of migrating (*p <* 0.05) (**Figures 4D, E**) and invading cells (*p <* 0.001) (**Figures 4F, G**) in the FN1-Ab group.

### 3.5 The FN1-ITG-PI3K/AKT axis promotes invasion and migration of pancreatic cancer cells

**Figure 5:**
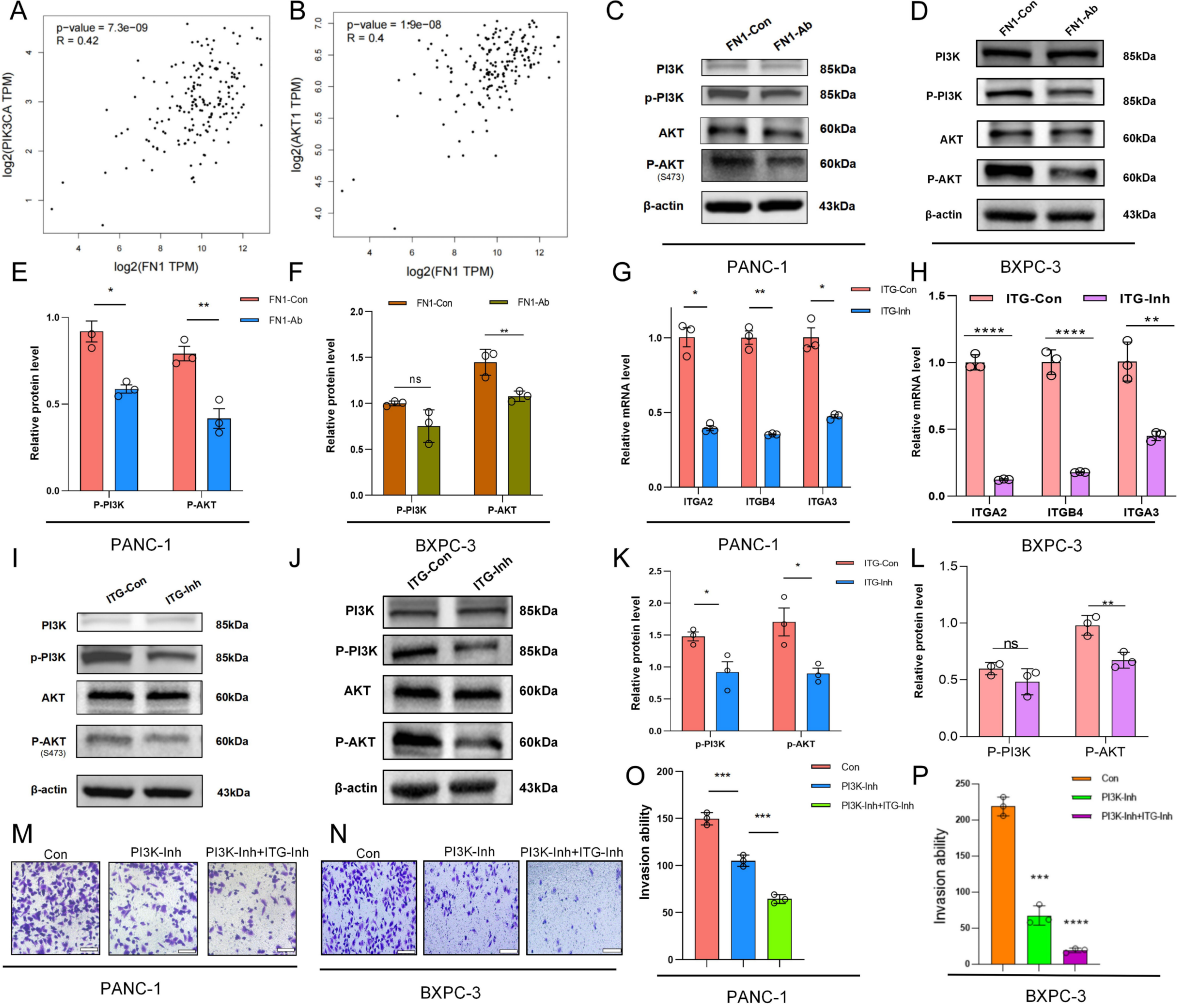
The FN1-ITG-PI3K/AKT axis promotes invasion and migration of pancreatic cancer cells. **(A, B)** GEPIA database analysis revealing a significant positive correlation between FN1 expression and the expression of key genes in the PI3K/AKT pathway (PIK3CA, AKT1). **(C, D)** Western blot results showing changes in phosphorylation levels of p-AKT and p-PI3K in the FN1-Ab group. The Western blot results demonstrated the changes in the phosphorylation levels of p-AKT and p-PI3K in the FN1-Ab groups of PANC-1 and BXPC-3 cells. **(E, F)** Statistical data corresponding to **(C, D)**. **(G, H)** mRNA expression changes of integrin genes (ITGA2, ITGB4, ITGA3) before and after treatment with an integrin inhibitor (ITG-Inh). The changes in mRNA expression of integrin genes *ITGA2*, *ITGB4*, and *ITGA3* in PANC-1 and BXPC-3 cells before and after treatment with the integrin inhibitor ITG-Inh. **(I, J)** Western blot results demonstrating changes in protein levels of p-AKT and p-PI3K in the ITG-Inh group compared to the control group (ITG-Con). The Western blot results indicated that in PANC-1 and BXPC-3 cells, compared with the control group (ITG-CON), the protein levels of p-AKT and p-PI3K in the ITG-Inh group were altered. **(K, L)** Statistical data corresponding to **(I, J)**. **(M, N)** Changes in invasion ability after combined inhibition of PI3K and integrins (PI3K-Inh + ITG-Inh). The changes in invasive capacity following the combined inhibition of PI3K and integrins (PI3K-Inh + ITG-Inh) in PANC-1 and BXPC-3 cells. **(O, P)** Statistical data corresponding to **(M, N)**.

Previous transcriptome and single-cell data suggested that FN1 is a core hub gene in the PI3K/AKT pathway, but its specific regulatory mechanism remained unclear. To verify the direct association between FN1 and the PI3K/AKT pathway, we analyzed the GEPIA database and found significant positive correlations between FN1 and key genes in the PI3K/AKT pathway (*PIK3CA*, *AKT1*) (Spearman coefficients R=0.42 and 0.40, *p <* 0.05) (**Figures 5A, B**). To further elucidate the molecular mechanism, we added an FN1 neutralizing antibody (FN1-Ab) to the co-culture model, and Western blot results showed significantly reduced phosphorylation levels of p-AKT (*p <*0.05) and p-PI3K (*p <*0.01) in the FN1-Ab group compared to the control group (FN1-Con) (**Figures 5C, D**), suggesting that FN1 drives invasion by activating the PI3K/AKT pathway. However, as an ECM protein, FN1 signals through cell surface receptors. Based on known interactions between FN1 and the integrin family (*ITGA2*, *p <*0.05; *ITGB4*, *p <*0.01; *ITGA3*, *p <*0.05), we hypothesized that integrins are key mediators in the FN1-PI3K/AKT axis. To test this hypothesis, we treated the co-culture system with an integrin inhibitor (ITG-Inh) and found significant downregulation of integrin genes (*ITGA2*, *ITGB4*, *ITGA3*) in the ITG-Inh group (**Figure 5E**). Western blot results showed significant decreases in the protein levels of p-AKT (*p <* 0.05) and p-PI3K (*p <* 0.05) in the ITG-Inh group (**Figures 5F, G**). To further assess the synergistic effect of combined targeting, we inhibited both PI3K and integrins (PI3K-Inh + ITG-Inh) and found a further decrease in invasion ability compared to single-drug groups (*p <* 0.01) (**Figures 5H, I**). These data indicate that FN1 activates the PI3K/AKT pathway through integrin receptors, and combined inhibition can synergistically block this signaling axis, providing new insights for clinical treatment.

Both preliminary transcriptomic and single-cell data have suggested that FN1 serves as a core hub gene in the PI3K/AKT signaling pathway, although its specific regulatory mechanism remains unclear. To validate the direct association between FN1 and the PI3K/AKT pathway, we analyzed the GEPIA database and found a significant positive correlation between the expression of FN1 and key genes in the PI3K/AKT pathway (PIK3CA, AKT1) (Spearman’s correlation coefficient R=0.42 and 0.40, respectively, p < 0.05) (**Figures 5A, B**). To further elucidate the molecular mechanism, we established co-culture models using PANC-1 and BXPC-3 cells and added an FN1 neutralizing antibody (FN1-Ab). Western blot results demonstrated that the phosphorylation levels of p-AKT and p-PI3K in the FN1-Ab groups of both PANC-1 and BXPC-3 cells were significantly reduced compared to the control groups (FN1-Con) (p < 0.01) (**Figures 5C–F**), suggesting that FN1 drives invasion by activating the PI3K/AKT pathway. However, as an ECM protein, FN1 requires cell surface receptors to transmit signals. Based on the known interactions between FN1 and integrin family members (such as ITGA2, ITGB4), we hypothesized that integrins are key mediators of the FN1-PI3K/AKT axis. To test this hypothesis, we treated the co-culture systems with an integrin inhibitor (ITG-Inh). We observed a significant downregulation in the mRNA expression of integrin genes (*ITGA2, ITGB4*, *ITGA3*) in the ITG-Inh groups of both PANC-1 and BXPC-3 cells (**Figures 5G, H**). Western blot results further confirmed that the protein levels of p-AKT and p-PI3K were significantly decreased in the ITG-Inh groups (p < 0.001) (**Figures 5I–L**). To further assess the synergistic effects of combined targeting, we simultaneously inhibited PI3K and integrins (PI3K-Inh + ITG-Inh) and found that the invasive capacity in the combined group was further reduced compared to the monotherapy groups (p < 0.001) (**Figures 5M–P**). These data indicate that FN1 activates the PI3K/AKT pathway through integrin receptors, and that combined inhibition can synergistically block this signaling axis. The synergistic effects of combined targeting are not specific to PANC-1 cells alone and provide new insights for clinical treatment strategies.

### 3.6 High FN1 expression is associated with poor prognosis and an immunosuppressive microenvironment

**Figure 6:**
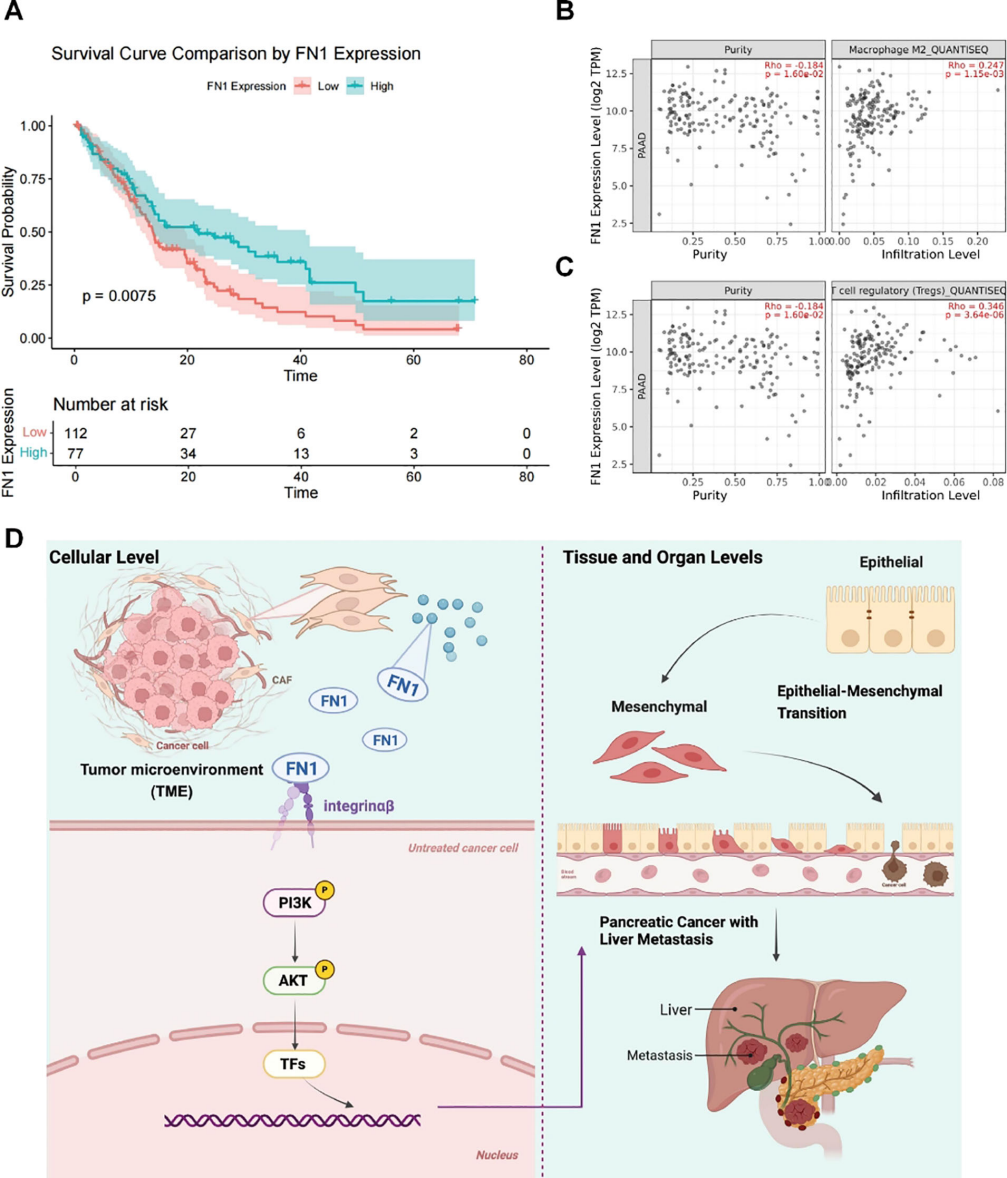
High FN1 expression is associated with poor prognosis and an immunosuppressive microenvironment. **(A)** Clinical data analysis showing the relationship between FN1 expression and patient survival. **(B)** TIMER2.0 displaying the correlation between FN1 expression and M2 macrophage infiltration in pancreatic cancer (PAAD). **(C)** TIMER2.0 showing the correlation between FN1 expression and Treg cell infiltration in PAAD. **(D)** Mechanism diagram illustrating how FN1 activates the PI3K/AKT pathway by binding to integrin receptors, thereby promoting the invasion and metastasis of pancreatic cancer cells.

Clinical data showed that patients with high FN1 expression had significantly shorter survival times (**Figure 6A**) and that FN1 expression was positively correlated with infiltration of M2 macrophages and Treg cells (**Figures 6B, C**). This result links the molecular mechanism to clinical outcomes and the immune microenvironment, suggesting that targeting FN1 may simultaneously inhibit metastasis and reverse immune suppression. The mechanism diagram further clarifies that FN1 activates the PI3K/AKT pathway by binding to integrin receptors, thereby promoting the invasion and metastasis of pancreatic cancer cells.

## 4 Discussion

The high metastatic potential of pancreatic cancer is closely associated with the dynamic remodeling of the TME, wherein CAFs drive the metastatic process by secreting ECM components and signaling molecules. Through the integration of multi-omics data, functional experiments, and clinical analyses, this study systematically unveils the molecular mechanism by which CAF-derived FN1 promotes pancreatic cancer metastasis via the integrin-PI3K/AKT axis and elucidates, for the first time, its interaction with an immunosuppressive microenvironment. These findings aim to provide a novel theoretical basis for the treatment of pancreatic cancer.

Transcriptome analysis revealed that differential genes were significantly enriched in ECM-receptor interaction, focal adhesion, and the PI3K/AKT pathway (**Figures 1C, D**), suggesting the synergistic role of ECM remodeling and PI3K/AKT signaling in pancreatic cancer metastasis. Notably, based on CytoHubba analysis, FN1 was identified as the hub gene with the highest interaction score in the PI3K pathway (**Figures 1E, F**). This discovery aligns with previous studies indicating that FN1 activates pro-survival signals through integrin receptors in various cancers (15, 16). However, this study further localized the specific high expression of FN1 in CAF subpopulations through single-cell sequencing (**Figure 3E**), revealing its functional specificity at spatial resolution. This echoes the research by Zhan Y et al., where metastatic CAFs promote colorectal cancer metastasis by secreting ECM components such as FN1 and COL1A1 (12). The novelty of this study lies in identifying FN1 as the core driving molecule in the PI3K/AKT pathway.

Functional experiments demonstrated that TGF-β-activated CAFs secrete FN1, significantly upregulating IL-6, IL-8, and MMP2 (**Figure 4B**). These factors not only reshape the ECM structure but also recruit immunosuppressive cells (17). By neutralizing FN1 or inhibiting integrin receptors, the invasive capacity of pancreatic cancer cells was significantly reduced (**Figures 4F, G**, **5M, N**), with combined inhibition further decreasing invasiveness (**Figures 5M, N**). Western blot analysis confirmed that FN1 activates the PI3K/AKT pathway through integrin receptors (**Figures 5I, J**). This mechanism aligns with the “integrin-ECM mechanical signal axis” model proposed by the Chastney MR team (18), but this study is the first to reveal the synergistic effect of the PI3K/AKT signaling pathway and integrins in pancreatic cancer and propose a combined targeting therapeutic strategy.

In addition to regulating prometastatic signals, this study is the first to find a significant positive correlation between high FN1 expression and the infiltration of M2 macrophages and Tregs (**Figures 6B, C**). M2 macrophages inhibit antitumor immunity by secreting IL-10 and TGF-β (19), while Tregs suppress T-cell function through immune checkpoints such as CTLA-4 (20). Therefore, FN1 may mediate immunosuppression through two mechanisms: (1) as an ECM scaffold, its rigid structure provides physical residence sites for immunosuppressive cells; (2) by upregulating PD-L1 expression in tumor cells through integrin signaling, indirectly inhibiting T-cell activity (21). This discovery expands FN1’s traditional role from a “passive ECM component” to an “active immune regulatory factor,” revealing its crucial role in shaping the “cold tumor” microenvironment. Clinical survival analysis further supports the prognostic value of FN1, with shortened median survival in patients with high expression (**Figure 6A**), suggesting its potential as a biomarker.

Although drugs targeting the PI3K/AKT pathway have shown progress in breast cancer, their efficacy in pancreatic cancer is limited (22). This study suggests that combined inhibition of PI3K/AKT signaling and integrins can synergistically inhibit pancreatic cancer cell metastasis (**Figures 5M, N**), providing new insights for overcoming monotherapy resistance. However, translational applications still face challenges: (1) The high heterogeneity of CAFs may lead to differences in treatment response, necessitating the development of subpopulation-specific targeting strategies; (2) The upstream regulatory network of FN1 has not been elucidated; (3) The synergistic effect of FN1 and immune checkpoints needs further validation. Future research could explore the combined application of FN1 antibodies and immunotherapy using patient-derived organoids (PDOs) and orthotopic metastasis models.

## 5 Conclusion

pancreatic cancer metastasis through the integration of transcriptome data, single-cell transcriptome data, functional experiments, and clinical analyses. Firstly, single-cell sequencing identified the specific high expression of FN1 in metastatic CAF subsets, which directly drives EMT and cancer cell invasion via activation of the PI3K/AKT signaling pathway through integrin receptors. Secondly, the high expression of FN1 is significantly positively correlated with the infiltration of M2 macrophages and Treg cells, suggesting its indirect promotion of metastasis by shaping an immunosuppressive microenvironment. Clinical data analysis further confirms that patients with high FN1 expression have a significantly shorter survival period, supporting its potential as a prognostic biomarker. The innovation of this study lies in revealing the core role of the FN1-integrin-PI3K/AKT axis and proposing a combined therapeutic strategy targeting both the PI3K/AKT signaling pathway and integrins, providing a new avenue for overcoming therapeutic resistance in pancreatic cancer.

## Data Availability

The raw data supporting the conclusions of this article will be made available by the authors, without undue reservation.
