# Supplementary material for: FN1 from cancer-associated fibroblasts orchestrates pancreatic cancer metastasis via integrin-PI3K/AKT signaling
Source: Front Oncol. 2025 Jul 3;15:1595523. doi: 10.3389/fonc.2025.1595523 (PMC12267018; doi:10.3389/fonc.2025.1595523)
Supplement: Supplementary file 1 [file DataSheet1.docx]

Supplementary Material


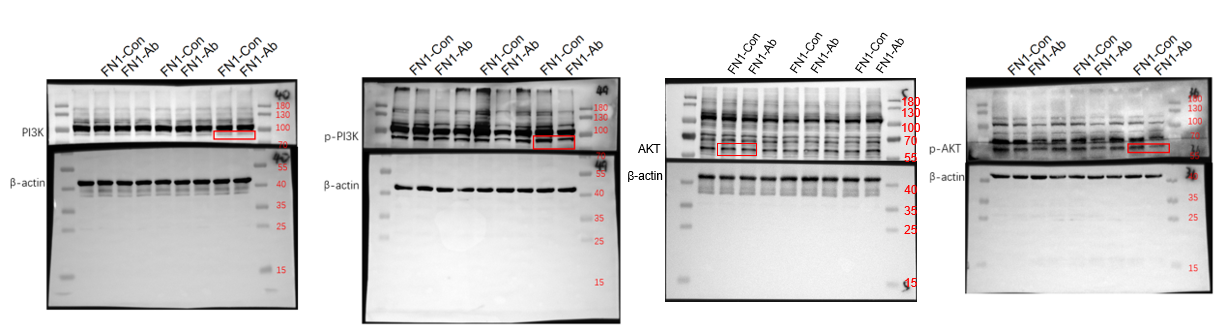


**Supplementary Figure 1.** Whole membrane of Figure 5C.

Each of our results was repeated 3 times, and what we present in the figure has been marked with a red box


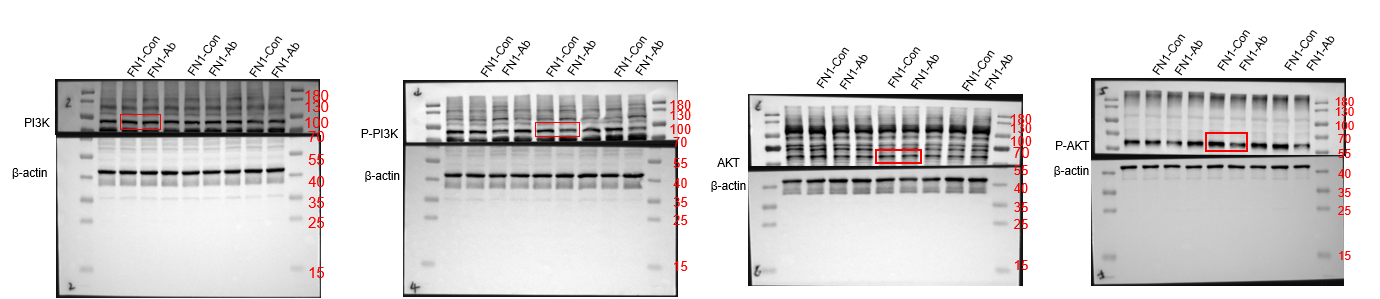


**Supplementary Figure 2.** Whole membrane of Figure 5D.

Each of our results was repeated 3 times, and what we present in the figure has been marked with a red box


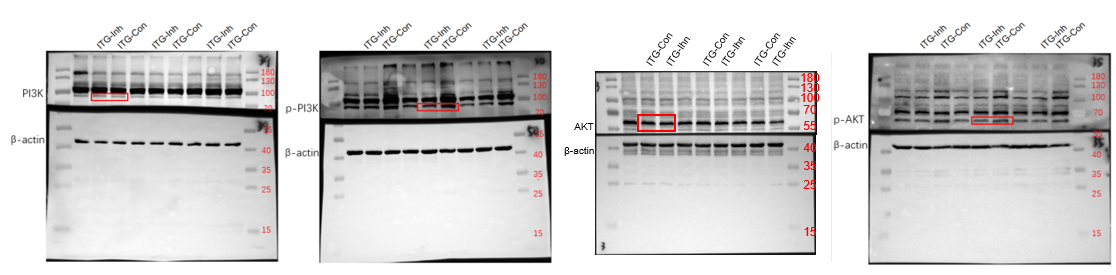


**Supplementary Figure 3.** Whole membrane of Figure 5I.

Each of our results was repeated 3 times, and what we present in the figure has been marked with a red box.


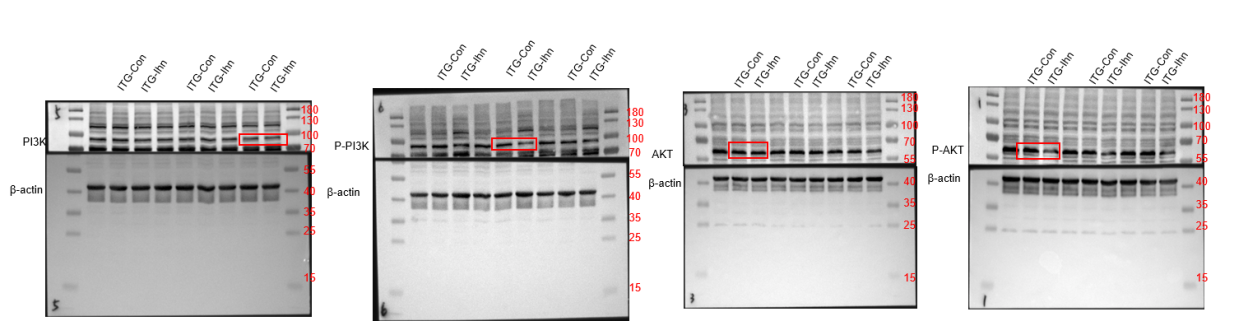


**Supplementary Figure 4.** Whole membrane of Figure 5J.

Each of our results was repeated 3 times, and what we present in the figure has been marked with a red box.
